# Visual and haptic cues in processing occlusion

**DOI:** 10.3389/fpsyg.2023.1082557

**Published:** 2023-03-10

**Authors:** Hiroshige Takeichi, Keito Taniguchi, Hiroaki Shigemasu

**Affiliations:** ^1^Computational Engineering Applications Unit, Head Office for Information Systems and Cybersecurity (ISC), RIKEN, Wako, Saitama, Japan; ^2^Open Systems Information Science Team, Advanced Data Science Project (ADSP), RIKEN Information R&D and Strategy Headquarters (R-IH), RIKEN, Yokohama, Kanagawa, Japan; ^3^School of Information, Kochi University of Technology, Kami, Kochi, Japan

**Keywords:** image segmentation, depth cues, visual pathways, virtual reality, haptic perception

## Abstract

**Introduction:**

Although shape is effective in processing occlusion, ambiguities in segmentation can also be addressed using depth discontinuity given visually and haptically. This study elucidates the contribution of visual and haptic cues to depth discontinuity in processing occlusion.

**Methods:**

A virtual reality experiment was conducted with 15 students as participants. Word stimuli were presented on a head-mounted display for recognition. The central part of the words was masked with a virtual ribbon placed at different depths so that the ribbon appeared as an occlusion. The visual depth cue was either present with binocular stereopsis or absent with monocular presentation. The haptic cue was either missing, provided consecutively, or concurrently, by actively tracing a real off-screen bar edge that was positionally aligned with the ribbon in the virtual space. Recognition performance was compared between depth cue conditions.

**Results:**

We found that word recognition was better with the stereoscopic cue but not with the haptic cue, although both cues contributed to greater confidence in depth estimation. The performance was better when the ribbon was at the farther depth plane to appear as a hollow, rather than when it was at the nearer depth plane to cover the word.

**Discussion:**

The results indicate that occlusion is processed in the human brain by visual input only despite the apparent effectiveness of haptic space perception, reflecting a complex set of natural constraints.

## 1. Introduction

Occlusion is a typical problem in image processing that involves separating regions that correspond to objects that are apart in the external three-dimensional (3D) space but adjoined in the projected two-dimensional (2D) image because of the proximity of the lines of sight. The occlusion problem comprises two subproblems. First, contours in an image must be segmented. Proper segmentation cannot be obtained by tracking contours in the projection because the contours that are separated in the 3D space may be misleadingly connected in the 2D projection. Second, the segmented contours must be completed to fill in the gaps such that the completed contour is a good estimation of the projected contour without occlusion, that is, occlusion-invariant. Occlusion remains a difficult problem in computer vision (Garcia-Garcia et al., 2017; Albalas et al., 2022). While the input to the second problem of interpolation comprises the output from the first problem of segmentation, the first problem recursively depends on the output from the second problem (Takeichi et al., 1995). For example, if a face is partly occluded and the missing parts are to be completed, one must first know whether there is actually a face without recognizing it. The question is how much of the segmentation can be addressed without committing to interpolation.

The depth relationship appears to be an independent and effective cue for segmentation in general. Segmentation can be significantly changed by inverting the depth relationships between overlapping figures (Nakayama et al., 1990). However, when a stronger cue than the depth is available, depth may not affect the segmentation. For example, the effect of stereoscopic depth is reduced or lost when visual motion is also provided as a segmentation cue (Takeichi, 1999). The problem of segmentation may be solved based on the shape of the contour without occlusion. Completion in occlusion, which is called amodal completion in experimental psychology, may be most parsimoniously predicted by the shape of completed figures (van Lier et al., 1995) in 3D (Tse, 1999, 2017; Kellman et al., 2005). Although dependence on 3D shape apparently supports the iterative computation between segmentation and interpolation, segmentation can still be one-shot if it considers the curvature-based geometric relationship between the 3D shape and its 2D projection (Richards et al., 1987; Elder, 2018). In fact, human perception shows sensitivity to curvature, which is invariant under the projection from 3D to 2D, in processing occlusions (Takeichi, 1995).

Simple segmentation by depth may be performed at an early level in visual processing before the two cortical streams, one for object identity and the other for spatial relationships or interactions (Ungerleider and Mishkin, 1982; Goodale and Milner, 1992), diverged as the ventral and dorsal pathways in the primate visual system (Bakin et al., 2000; O'Herron and von der Heydt, 2013). However, completion that concords with the spatial arrangement of several multipart objects in naturalistic scenes (Tse, 1999, 2017; Kellman et al., 2005) may imply that processing occlusion requires a significant interaction between the two parallel visual pathways. The visual system may not compute the connectedness likelihood only through a simple measure such as simplicity (Buffart and Leeuwenberg, 1981), relatability (Kellman and Shipley, 1991; Kellman et al., 2005) or curvature (Takeichi et al., 1995) but a set of constraints (DiMattina et al., 2012). Tse’s (2017) demonstration of amodal completion of fluids or slimy objects further implies that complexity of the constraints may be comparable to “naive” or intuitive physics, such as viscosity, cohesiveness, and specific gravity of fluid and gravitational force. On the one hand, it is natural because processing occlusion is scene analysis. The relevant constraints may span from optics such as the generic view principle (Freeman, 1994; Kitazaki and Shimojo, 1996; Albert, 2001) to laws of mechanics that predict probable and improbable shape, deformation and structure. On the other hand, it also implies that a wide variety of brain areas, particularly those of multisensory integration, may be involved in processing. Material properties that can be related to deformation are estimated in the ventral pathway (Goda et al., 2014), while arrangements and mechanical relationships between several such objects must involve processing in the dorsal pathway. If the perceived property of fluid needs to be integrated with the perceived spatial layout of scattered clusters of such fluid together with the potential occluder to estimate connectedness likelihood in reference to intuitive physics, then the ventral and dorsal pathways must interact as such (c.f. Van Dromme et al., 2016). In addition, whereas it can be hardly tested empirically whether or not the visual input alone is enough for the development of intuitive physics in visual perception, it is also difficult to imagine how concepts such as weight and force develop in visual modality without any reference to tactile or haptic inputs. In fact, perceived occlusion is a purely visual phenomenon because occlusion is defined as interruption of the line of sight. However, it sounds odd if the visual system uses an internal model of intuitive physics to solve the problem only in the visual modality because the intuitive physics itself is likely to be acquired through interaction between visual and tactile or proprioceptive modalities. If processing occlusion is based on intuitive mechanics, then knowledge from previous haptic input, which is the basis of intuitive mechanics, may be used in processing occlusion.

In this study, we investigated the cues that are or are not used in perceptual segmentation and the completion of partially occluded figures. Letters were used as stimulus figures. In the experiment, word recognition performance was compared in the presence and absence of visual and haptic cues to the depth of the occluder. If information provided by haptic input aids in the recognition of partially occluded letters when solving the occlusion or segmentation problem, the presence of haptics may provide information regarding the relationship between the occluded letters and the source of the occlusion. Alternatively, if the effect of haptic input is limited to depth perception, this may imply that haptic inputs have limited roles in recognizing partially occluded letters. The effectiveness of the visual and haptic cues was also evaluated through depth judgments and confidence ratings of the judgments. Confidence was measured because it can be sensitive to potential cue effectiveness (Fairhurst et al., 2018).

## 2. Methods

A word recognition experiment was performed to examine perceptual completion using virtual reality (VR). Word recognition performance was assessed when the central part of the word stimulus was masked by a horizontal virtual ribbon. The virtual reality experiment was conducted with 15 students as participants. The participants had to fill in the missing central part by connecting the top and bottom parts that remained in the visual stimulus to recognize the words. The edges of the ribbon were implicit and invisible, as if the ribbon were camouflaged to have the same lightness, color, and texture as the background. Cues to the depth of the ribbon were provided visually through random dots scattered over the surface of the ribbon, haptically by active tracing of the edge of the ribbon, or both. This unnatural occlusion was simulated to reduce visual cues. Visual input is provided as a stream of two-dimensional arrays of pixels, i.e., images, while haptic input is provided as a time-series of points by scanning the target over time. Because it is difficult to control two-dimensional haptic exploration to give comparable inputs in both visual and haptic modalities, the haptic input was limited to the edges. The visual input was thus similarly limited to the edges with the obscure occluder.

### 2.1. Ethics statement

Data collection and processing were performed in accordance with the principles of the Declaration of Helsinki. The protocols of the human experiments in this study were approved prior to initiation by the institutional review board of Kochi University of Technology (138-C2) and Wako Third Ethical Committee of RIKEN (Wako3 2020-27).

### 2.2. Participants

A total of 15 volunteers (13 men and 2 women; 22.06 years old with a standard deviation of 0.92 years) participated in the experiment. They were students at Kochi University of Technology or their affiliates. All participants had normal or corrected-to-normal vision and normal binocular stereopsis, which was confirmed with the experimental setup before the experiment, and were native Japanese readers. Written informed consent was obtained from each participant before participation.

### 2.3. Stimulus and apparatus

**Figure 1:**
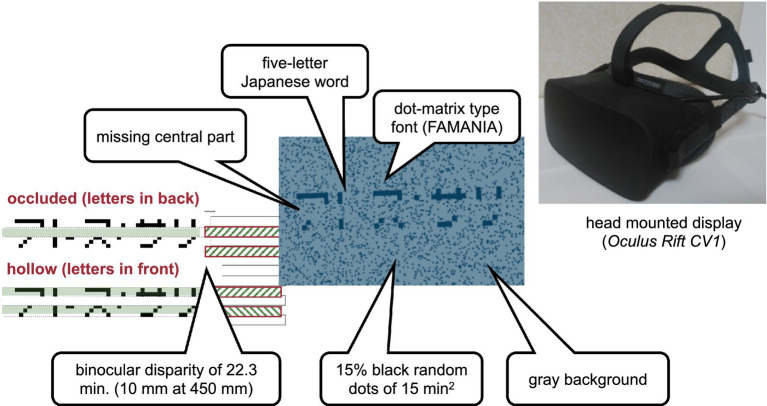
Visual stimulus. The haptic stimulus (Figure 2) was a wooden board. Four horizontal square-wave bumps were created on the board by gluing square columns in parallel onto the surface. Multiple bumps were made to change the physical position of the edge across trials to eliminate artifactual cues and interferences from a nonvarying stimulation. The edges of the square columns were aligned with the edges of the virtual ribbon in the VR space. The participants actively traced one of the edges of one of the columns using the tip of the index finger of their right hands to receive haptic input (Figure 3). An Oculus Touch controller was used for positional alignment between vision and haptics. The participants held the Oculus Touch controller while tracing the specified edge. The position of the fingertip was visually indicated by a blue virtual ball that moved in real-time synchrony with the motion of Oculus Touch in the head-mounted display. The VR system was implemented using Unity 2017.4.15f1. The latency was within 25 ms after the movement onset and then within 5 ms on average (Warburton et al., 2022). The participant sat on a chair and was confronted with a wooden board on a desk 45 cm in front of them. A chinrest was used to minimize head movement.

A visual-haptic multimodal stimulus was presented as a type of mixed reality. The visual stimulus (Figure 1) comprising words for visual recognition and a horizontal virtual ribbon that masked the central part of the word was presented on a head-mounted display (Oculus Rift CV1). The spatial resolution of the head-mounted display was 1,080 by 1,200 per eye, and the diagonal field of view was approximately 110 degrees. The refresh rate was 90 Hz. The virtual ribbon simulated an occlusion when it appeared to be above and covering the central part of the word or a hollow when it appeared to be farther in depth at the central part of the word. The word stimuli were five-letter Japanese words written in katakana characters, which are alphabet-like phonograms in Japanese. The words were obtained from an open database for teaching Japanese as a second language and, therefore, were quite commonly used by native readers. A total of 337 five-letter words were extracted from the database, excluding words containing one or more small characters to indicate the palatalized “y” sound or double consonants in Japanese. The characters were displayed using public-domain font-type FAMania for the ease of camouflage, which mimics low-resolution (7 × 7 pixels) characters that were used on gaming PCs during the 1980s. The ribbon was positioned such that its edges naturally coincided with pixel boundaries, that is, between the first and the second rows and between the sixth and the seventh rows of the pixel matrix. If a font type with a higher resolution was used, placing the virtual ribbon would inevitably introduce conspicuous linearly aligned terminators, which could be a strong cue to depth discontinuity, such as abutting gratings that induce perception of illusory contours (Soriano et al., 1996). The background was gray, with scattered black random dots of 15% density. The random dots had a binocular disparity of 22.3 min in the conditions with binocular cues to depth.

**Figure 2:**
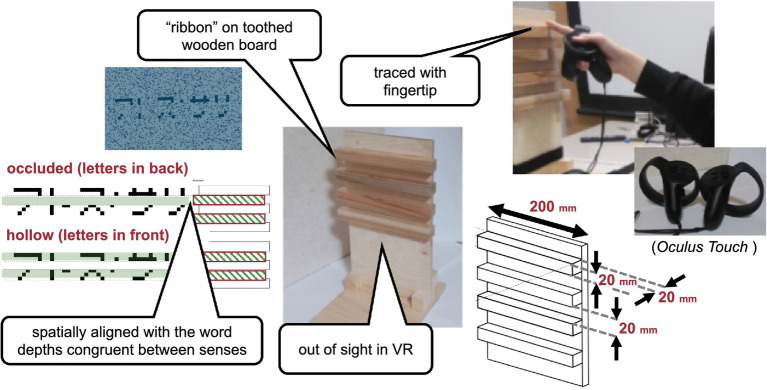
Haptic stimulus.

**Figure 3:**
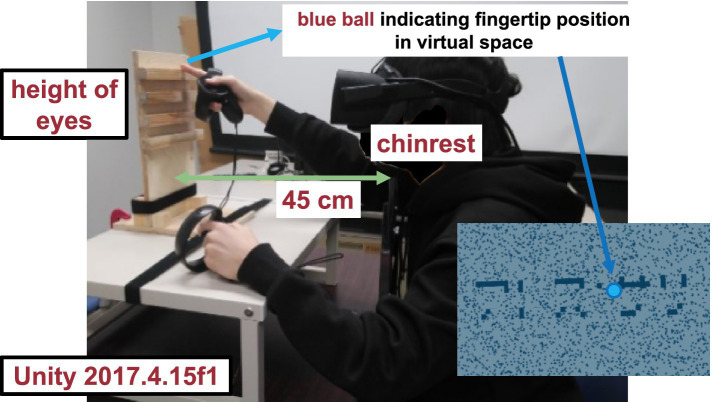
Experimental setup.

### 2.4. Design and procedure

In each trial, a word was presented with the virtual ribbon. The task was to verbally report the word recognized after stimulus presentation. There were 12 combinations of two levels of depth, two types of visual cues, and three types of haptic cues. The depth of the word was either in the background of the masking virtual ribbon that appeared to be an occlusion or in front of the virtual ribbon that appeared to be hollow. The visual cue was either present as a horizontal binocular disparity of the dots in the area covered by the virtual ribbon or absent by monocular presentation with a blank screen in the other eye. The haptic cue was either absent, consecutive, or concurrent. When the haptic cue was absent, the visual stimulus (i.e., the word and the virtual ribbon) was presented for 10 s, with the ribbon as an occlusion or a hollow, and with or without binocular disparity, followed by a uniform gray screen for another 10 s. The participant was instructed to report the recognized word verbally during the latter 10 s. When the haptic cue was concurrent, the participants were additionally instructed to actively and concurrently trace one of the edges of the ribbon, but not the word, with the fingertip during the former 10 s period simultaneously with visual presentation. When the haptic cue was consecutive, a pair of red balls was first visually presented against a uniform gray background before the visual presentation of the word. The red balls indicate the positions of the ends of the edge in the VR space. The participants were instructed to actively trace the camouflaged edge with the fingertip for the first 5 s and were instructed to take the finger off the board during the following 10 s period for visual presentation. Thus, the edge was not traced when the haptic cue was absent, was traced simultaneously with the visual presentation when the haptic cue was concurrent, and was traced only during the first 5 s without visual stimulus but not during the following 10 s with the visual stimulus when the haptic cue was consecutive.

Each condition was presented in a block of five trials, in which five different words were presented. The 12 conditions were presented in a set of 12 blocks in randomized order. Each participant performed four sets on 2 days. Therefore, there were 240 trials for each participant. Each condition was repeated 20 times in four sets of five blocked trials. The same set of words was used for all participants. Different words were used in different trials so that the same word was used only once for each participant. Different words were randomly assigned to various conditions across the participants. The eye stimulated for the monocular presentation alternated between successive blocks of the monocular conditions. The participants traced the upper or lower edge of the ribbon with the palm facing downward or upward, respectively, in the two halves of the blocks in the occlusion condition. Similarly, they traced the upper or lower edge of the ribbon with the palm facing upward or downward, respectively, in the two halves of the blocks in the hollow condition. The edge to be traced and direction of the palm were varied to eliminate the potential association between the hand shape and depth as unintended cues. The potential association could provide information regarding the depth artifactually and, therefore, could contaminate the results. The hand shape was specified by instructions for each block and alternated between successive blocks of consecutive and concurrent haptic cue conditions. The edge to be traced varied randomly across trials among the three alternatives with different heights to minimize the potential effect of position in the visual field or peri-personal space. The participants also had to report the perceived depth with confidence on a seven-point scale ranging from −3 to 3 at the end of each block. The confidence as well as the perceived depth was measured in order to identify the extent to which there was an effect of haptic input: whether the haptic input does not influence perceived depth at all or it does influence perceived depth but not recognition. The participants could take breaks at will at any block interval.

The participants’ head position was continuously monitored by a computer, and a trial was aborted if the computer detected a displacement larger than 30 mm away from the original position. The same condition was then conducted later in an additional trial to fill in the missing observation. The participants were informed of the constraint during the instruction. The threshold of 30 mm was empirically determined such that any intentional head movement was detected, which resulted in the participant’s voluntary immobilization and few aborted trials. Therefore, although there could be some influence of head movement and motion parallax because the stimuli were not presented statically regardless of the participants’ head position, our method must have led to the most natural and cost-effective suppression of potential artifacts of head motion and motion parallax.

### 2.5. Statistics

The rate of correct word recognition was calculated for each of the 12 conditions for each of the 15 participants across 20 repetitions. The stimulus word was considered correctly recognized (score = 1/20) if the response fully matched the five-letter stimulus word and not (score = 0/20) otherwise. The rate of correct depth perception was calculated but not evaluated because it was saturated by the ceiling. The mean confidence rating was calculated and evaluated as the mean of the absolute value of the perceived depth on the seven-point scale for each of the 12 conditions for each of the 15 participants across the four repetitions. A three-way analysis of variance was performed with repeated measurements using Anova-kun 4.8.5 (Iseki, 2020) on R 4.0.2 (R Core Team, 2018) for each of the recognition rate and the mean depth confidence rating. The factors were depth, visual cues, and haptic cues: Depth was either letters-in-front or letters-in-background; the visual cue was either present through binocular stereopsis or absent through monocular presentation; and the haptic cue was absent, consecutive, or concurrent. Multiple comparisons were performed using Shaffer’s method for the corrected alpha of 0.05 for individual ANOVA of the two different indices: recognition rate and depth rating. Violations to sphericity were tested using Mendoza’s multisample sphericity test. In addition, to examine the effects of deviation from the normal distribution regarding the word recognition rate, a mixed model analysis was performed assuming a binomial in place of normal distribution after transformation by a logistic function.

The general linear mixed models included fixed factors of depth, visual cue, haptic cue and all their second- and third-order interactions and random factors of participant and position of the three-alternative traced edge. The analyses with general linear mixed models were performed using MATLAB R2021a or later.

### 2.6. Additional analyses

It is worth noting how much of the recognition performance measured in this task reflected the success of the amodal completion process. Some characters might be too hard or even impossible to identify due to occlusion of a critical part. In such cases, amodal completion would not necessarily contribute to correct answers. Thus, the same general linear mixed model was tested with the data after removing letters that were deemed too difficult to complete, as follows. First, the mean correct response rate was calculated for each of the 67 characters regardless of the condition or the participant. Second, the characters with relatively poor correct responses, namely, <0.8, were identified. Finally, the scores were recalculated based only on the remaining “easy” characters that could be recognized in more than 80% of the cases to be examined.

Responses with long latencies up to 10 s were allowed to accommodate the time that the participants needed, especially in the concurrent haptic cue condition, in which they performed a dual task. However, because responses with longer latencies are generally based more on cognitive processes, an analysis that is limited to data with shorter latencies may focus more on perceptual processes. Thus, word recognition was also evaluated when the data were limited to trials with response times shorter than the overall mean response time.

Each word was presented only once for each participant. However, potential differences between characters need further consideration. Because the order of the words was different between participants, different characters appeared at different positions along the progression of the experiment. Therefore, potential character-specific learning could have resulted in either a spurious bias, i.e., false positives, or extraneous variability that may contribute to error variance, i.e., false negatives. To rule out these potential artifacts, a measure of character-specific learning was constructed. For each of the 5 characters in the word stimulus, the frequency of appearance in all preceding stimuli was enumerated and summed up to be a “familiarity” index of the word for each trial of each participant. The familiarity index was added to the predictors in the analysis of the potential effects of perceptual learning. The last two analyses additionally included a random factor of interaction between position of the traced edge and trial number.

## 3. Results

**Figure 4:**
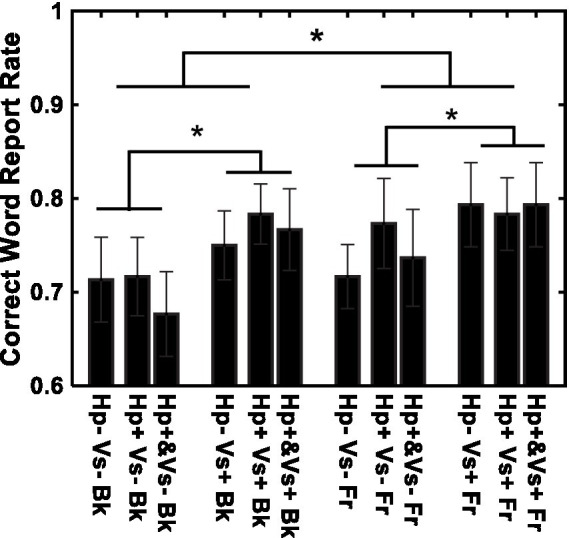
Recognition performance for individual conditions. *Vs*+: visual cue present with binocular stereopsis. *Vs*−: visual cue absent with monocular presentation. Hp+&: haptic cue to visual presentation. Hp+: haptic cue subsequently to visual presentation. Hp−: no haptic cue. Fr: virtual ribbon in front of the word. Bk: virtual ribbon as a hollow the word in the background. *: difference statistically significant.

Figure 4 shows the results of word recognition. The correct word-report rate is shown for each of the 12 conditions. Recognition was better in the visual cue present (*Vs*+) condition than in the visual cue absent (*Vs*-) condition [*F* (1, 14) = 11.51, *p* < 0.01, *η*^2^ = 0.027] and in the letters-in-front (Fr) condition than in the letters-in-background (Bk) condition [*F* (1, 14) = 5.13, *p* < 0.05, *η*^2^ = 0.008]. The type of haptic cue (Hp+&, Hp+, and Hp−) did not have a significant effect [*F*(2, 28) = 0.88, *p* = 0.45, *η*^2^ = 0.003]. None of the interactions were significant [Haptic-cue and depth: *F* (2, 28) = 0.18, *p* = 0.83, *η*^2^ = 0.003; Haptic-cue and Visual-cue: *F* (2, 28) = 0.53, *p* = 0.59, *η*^2^ = 0.001; Visual-cue and depth: *F* (1, 14) = 0.27, *p* = 0.60, *η*^2^ = 0.000; Visual-cue, Haptic-cue and depth: *F* (2, 28) = 1.12, *p* = 0.33, *η*^2^ = 0.003]. No violations to the sphericity test were found. The results were essentially the same when a linear mixed model was evaluated with a binomial distribution and the logistic link function. The effect of depth was *F* (1, 168) = 4.961, *p* = 0.02725, Cohen’s *f *^2^ = 0.03018 and *F* (1, 168) = 4.992, *p* = 0.02679, *f *^2^ = 0.02707 assuming a normal distribution and a binomial distribution, respectively. The effect of the visual cue was *F* (1,168) = 15.82, *p* = 0.00001033, *f *^2^ = 0.09624 and *F* (1,168) = 15.94, *p* = 0.00009746, *f *^2^ = 0.07766 assuming a normal distribution and a binomial distribution, respectively.

Whereas the criterion of 80% correct recognition was arbitrary, it separated 44 “easy” characters from 23 “difficult” characters, which were “クグシゼソゾタダチヅネハパビフプベペメヤユヨラ.” As a result of the analysis of the score that was recalculated only on the 44 easy characters with general linear mixed models, the effects of depth and visual cue remained the only significant factors [*F* (1,168) = 4.785, *p* = 0.03009, *f *^2^ = 0.02909; *F*(1,168) = 14.40, *p* = 0.0002061, *f *^2^ = 0.08755]. The effect of the haptic cue reached closer to the significance level [*F* (2,168) = 2.065, *p* = 0.1301, *f *^2^ = 0.02510]. As a result of the general linear mixed model that included the familiarity index as a fixed factor, the effect of the familiarity index of character-specific learning was highly significant [*F* (1,3576) = 48.84, *p* < 0.00001, *f *^2^ = 0.007416]. The only significant interaction with the familiarity index was with the visual cue [*F* (1,3576) = 5.015, *p* = 0.02519, *f *^2^ = 0.001344].

**Figure 5:**
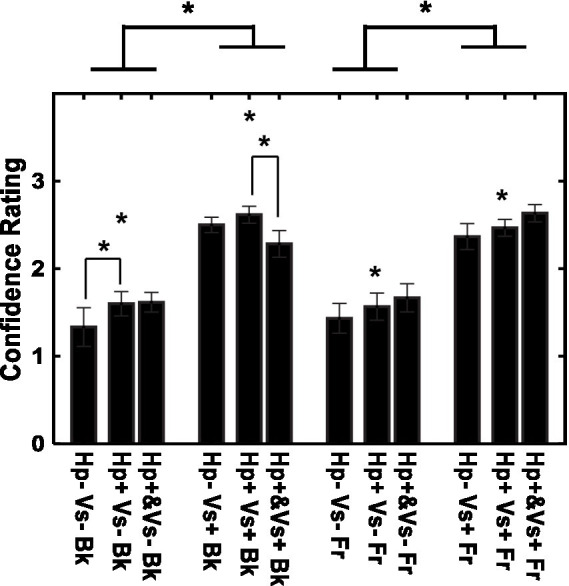
Confidence in depth perception for individual conditions. *Vs*+: visual cue present with binocular stereopsis. *Vs*−: visual cue absent with monocular presentation. Hp+&: haptic cue with visual presentation. Hp+: haptic cue subsequently to visual presentation. Hp−: no haptic cue. Fr: virtual ribbon in front of the word. Bk: virtual ribbon as a hollow in the word in the background. *: difference statistically significant.

There were fewer errors in depth judgments with the visual cue (21.11% of the trials) than in depth judgments without the visual cue (49.72%). The confidence in the depth rating is shown for each of the 12 conditions in Figure 5. The rating was more confident in the visual cue present condition than in the visual cue absent condition [*F* (1, 14) = 60.60, *p* < 0.001, *η*^2^ = 0.575]. There was also an effect of the type of haptic cue [*F* (2, 28) = 4.84, *p* < 0.05, *η*^2^ = 0.014], and confidence was larger in the consecutive-haptic-cue condition than in the haptic-cue-absent and concurrent-haptic-cue conditions after corrections for multiple comparisons (Shaffer method, *p < 0.05*). Furthermore, the interaction between visual and haptic cues was significant [*F* (2, 28) = 6.17, *p* < 0.01, *η*^2^ = 0.006]. When the visual cue was absent, the participants had more confidence in the consecutive-haptic-cue condition than in the haptic-cue absent condition. When the visual cue was present, the consecutive-haptic-cue condition was better than the concurrent-haptic-cue condition.

**Figure 6:**
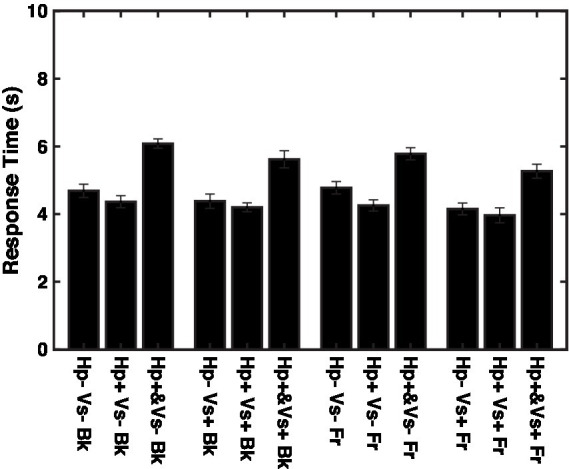
Response times for individual conditions. *Vs*+: visual cue present with binocular stereopsis. *Vs*-: visual cue absent with monocular presentation. Hp+&: haptic cue with visual presentation. Hp+: haptic cue subsequently to visual presentation. Hp−: no haptic cue. Fr: virtual ribbon in front of the word. Bk: virtual ribbon as a hollow in the word in the background.

If the data were limited to the trials with response times shorter than the overall mean response time, which was 4.7 s, then none of the effects were significant, except that the effect of the haptic cue was close to the significance level [*F* (2,1438) = 2.926, *p* = 0.05391, *f *^2^ = 0.004102]. The response time was longer in the concurrent haptic cue condition than in the other two haptic cue conditions, regardless of the depth and visual cue conditions (Figure 6, *F* (2,2517) = 162.8, *p* < 0.00001, *f *^2^ = 0.1304).

## 4. Discussion

Word recognition was better in the presence of binocular visual cues to depth but showed an atypical pattern of asymmetry in terms of the sign of the depth relationship; recognition performance was better in the letter-in-front condition than in the letter-in-background condition. Furthermore, the performance was not better in the presence of haptic cues to depth, although the participants reported higher confidence in depth perception with the presence of haptic cues. One interpretation of the atypical effect of the visual cue is that it enhanced but did not alter the segmentation based on the shape of the nonoccluded parts of the word stimuli. Another interpretation is that the occluder was made of sparse dots; therefore, there was less difference between the occluding surface and camouflaging texture in the background. One may be tempted to argue that the absence of a significant interaction between visual cue (*Vs*+ versus *Vs*−) and depth (Fr versus Bk) suggests an effect of binocular summation but not that of binocular stereopsis as the source of the effect of visual cue. Whereas better performance with the visual cue (*Vs*+ > *Vs*−) may indicate the effect of binocular summation, the pattern of the results is also consistent with the effect of binocular stereopsis. Namely, the difference between the depth conditions is larger with the visual cue (Hp−*Vs* + Bk versus Hp−*Vs* + Fr) than without the visual cue (Hp−*Vs*−Bk versus Hp−*Vs*−Fr) in the absence of interference from the haptic cues. The letter-in-front condition led to slightly better performance, probably because nearer stimuli are perceptually more salient. The apparent interference between the visual and concurrent haptic cues might have stemmed from the participant’s limited cognitive resources, such as attention or technical imperfections in the alignment between the visual and haptic presentations. Overall, the results suggest that perceptual completion is only visually depth-based and that haptic cues may not enhance segmentation.

### 4.1. Potential effects of cognitive factors and perceptual learning

The results were essentially the same when the scores were recalculated based only on “easy” characters that could be recognized in more than 80% of the cases. Some of the difficult characters are likely to be low-frequency characters in Japanese. For example, three characters “パプペ” among the 23 difficult characters have small circles at the top right corner to indicate voiceless “p” sound that mostly appears in loanwords.

There were no significant effects when the valid responses were limited to fast responses. However, it may not necessarily indicate that the present results merely reflect cognitive factors. The response times in the concurrent haptic cue condition were longer than those in the other two conditions. Thus, the division of data by response time effectively separated the data between conditions, thereby eliminating the differences by available cues. The longer response times in the concurrent haptic cue condition may be related to parallel processing of doubled information in a dual task rather than more top-down factors, as supported by the confidence in perceived depth. The depth seemed perceived more confidently with one or more cues than without cues but not in the concurrent haptic cue condition. Concurrent haptic processing seems to have interfered with visual processing when both cues were provided.

The result of the general linear mixed model with the familiarity index showed a highly significant effect of character-specific learning. However, the only significant interaction with the familiarity index was that with the visual cue, which suggested a decreasing effect of the visual cue as character-specific learning proceeded. It does not seem to have altered the potential effects of haptic cues in either way. Therefore, while character-specific learning took place, it does not seem to have altered the results.

### 4.2. Letter specificity

One potential reason why completion was shape-dominant rather than depth-dominant is that the figures to be completed were letters. Letters differ from other more general objects for computational and biological reasons. They are computationally 2D because they are not projections of 3D objects. Letter recognition is biologically exceptional in its automaticity, as demonstrated by the Stroop effect (Stroop, 1935) and specific and localized neural responses in several measurement modalities (Fujimaki et al., 1999; Cohen et al., 2000; Maurer et al., 2005). If this is the case, then different results could be obtained with various types of stimuli.

However, the absence of an effect of haptic input may still be related to occlusion being an inherently visual phenomenon. Processing occlusion may be only in the visual modality because occlusion is represented in the egocentric visual space that is explored with line of sight and not in the haptic space that is explored with points of touch. There is no occlusion in haptics because occlusion interrupts the line of sight, and there is no line of sight for haptics. Simultaneously, it has been reported that perception becomes haptic or tactile dominant when somatosensory input is more reliable than visual input (Ernst and Banks, 2002). If this is the case, haptic input might have some effects when the occlusion cannot be processed reliably by the generic view principle (Albert, 2001) because the vantage point is accidental and the visual input is severely limited.

### 4.3. Cortical site of processing occlusion

As the cortical area for perceptual completion of partially occluded letters is suggested by the results, area LO-1 is the best candidate for the neural correlate of the computation characterized through the experiment. Although the homology between human and nonhuman primate brains is not straightforward, the monkey counterpart of the human lateral occipital complex LOC (Grill-Spector et al., 2001) or the LO area in the ventral processing of object recognition is most likely the inferotemporal cortex, wherein the computation of occlusion-invariant representation is observed (Namima and Pasupathy, 2021). The lateral occipital area LO in the human brain can be divided into two subregions (Wandell et al., 2007), and subregion LO-2 shows greater shape selectivity than LO-1 (Silson et al., 2013; Vernon et al., 2016). As subregion LO-1 is adjacent to the human homolog of area V4, area LO-2 likely overlaps with area LOvt, which shows responsiveness to haptic input (Amedi et al., 2002; Monaco et al., 2017). Thus, the properties of the lower subregion LO-1 match shape processing without haptic input, suggesting that it is a good candidate for the area responsible for the perceptual completion of letters. Coactivation has also been reported between the LO area and the areas related to processing letters and words in the ventral occipitotemporal areas along the fusiform gyrus in the left hemisphere (Agrawal et al., 2020). LO-1 may be a good candidate considering the interaction between the dorsal and ventral pathways because LO-1 is closer to V3A/B, which belongs to the dorsal pathway, than LO-2. In fact, the vertical occipital fasciculus (VOF), or the fiber that connects the area LO and other ventral and lateral visual areas and the area V3A/B, has been identified in the human brain using a combination of fMRI, diffusion MRI and fiber tractgraphy (Takemura et al., 2017). Connections rather than individual areas may be more appropriate neural correlates of complex computations that are commonly found in human visual perception.

The present result may suggest unimodal three-dimensional representation as a precursor to multisensory three-dimensional representation. Constructing representation of the occluded part must be distinguished from recognizing the partly occluded or partly missing figures or objects. Computationally, recognition can be achieved without explicit representation, whereas explicit representation should help recognition. In other words, whereas explicit representation or completion is sufficient for recognition, it is not necessary. There must be some reason or computational benefit of actively constructing explicit representation or actively assuming presence rather than passively ignoring the absence of input from the invisible part. One such potential benefit is to predict the invisible part for future bodily interaction or tangibility. This may correspond to a bifurcation of the flow of information to the pathways for action and recognition (Goodale and Milner, 1992).

## 5. Conclusion

A VR experiment was conducted to investigate the effects of visual and haptic cues on recognizing partially occluded letters. Although visual cues enhanced letter recognition, enhancement was not specific to the sign of the depth relationships that are typical for occlusion. Haptic cues had no effect on recognition. The results suggest that LO-1 is the most likely cortical locus of the core for processing occlusion, although it must be examined in future studies whether the results are specific to letters or whether visual input dominates even when visual input is singular and haptic input provides comparatively more reliable or useful information. In either case, the present results illustrate how biological processing mirrors a complex set of natural constraints in processing occlusion.

## Data availability statement

The original contributions presented in the study are included in the article/Supplementary material, further inquiries can be directed to the corresponding author.

## Ethics statement

The studies involving human participants were reviewed and approved by Institutional Review Board of Koch University of Technology (138-C2) and Wako Third Ethical Committee of RIKEN (Wako3 2020-27). The patients/participants provided their written informed consent to participate in this study.

## Author contributions

HT and HS had a role in the conception, design, and interpretation of data. KT and HS had a role in the acquisition and analysis. HT and KT had a role in drafting the work. HS had a role in revising it critically. All authors contributed to the article and approved the submitted version.

## Funding

This study was supported by KAKENHI 20K03500 from the Japan Society for Promotion of Science.

## Conflict of interest

The authors declare that the research was conducted in the absence of any commercial or financial relationships that could be construed as a potential conflict of interest.

## Publisher’s note

All claims expressed in this article are solely those of the authors and do not necessarily represent those of their affiliated organizations, or those of the publisher, the editors and the reviewers. Any product that may be evaluated in this article, or claim that may be made by its manufacturer, is not guaranteed or endorsed by the publisher.
